# Patient needs and preferences for a kidney stone self-monitoring app: a pilot survey analysis

**DOI:** 10.1007/s00240-025-01900-3

**Published:** 2026-01-31

**Authors:** Duaa Tahir Mahmood, Helen L. Richards, Derek B. Hennessey

**Affiliations:** 1 School of Medicine, University College Cork, Cork, Ireland; 2 Department of Urology, Mercy University Hospital, Cork, Ireland; 3 Department of Clinical Psychology, Mercy University Hospital, Cork, Ireland

## Abstract

Nutritional habits play a role in the formation and recurrence of kidney stone disease (KSD). Dietary modification is important for the prevention of KSD recurrence. Mobile health (mHealth) applications (apps) may help patients manage their condition by offering personalised dietary guidance and tracking intake. This study assesses the interest of patients with recurrent kidney stone disease (KSD) in a self-monitoring app and identifies key features to guide its development. This cross-sectional quantitative study used an online survey to collect data from KSD patients. The survey collected information on patient demographics, healthcare smartphone usage, and preferences for app features. The findings will inform the development of an initial app prototype. Fifty-five patients participated (58.2% male; most aged 35–54). Kidney stone recurrence ranged from one episode (34.5%) to six or more (9.1%). Health app use varied: 29.1% used them daily, 36.4% never used them. While 30.9% were aware of relevant risk factors, most (61.8%) had only partial awareness. High concern about recurrence (65.5%) significantly predicted the likelihood of app use (*p* < 0.01). Interest in a prevention app was strong, with 72.8% indicating they were likely to use it. The likelihood of use was strongly correlated with recommending the app (*p* < 0.001), although recurrence frequency was not associated with interest. The top desired features included water tracking (81.1%), dietary advice (77.4%), educational content (56.6%), and diet tracking (56.6%). Participants prioritised hydration, diet, and personalised education, with 36.4% favouring tailored recommendations. This pilot survey highlights the strong interest of patients in a mobile health (mHealth) app for dietary self-monitoring in KSD. Larger studies, feasibility testing, and future trials will be needed to evaluate effectiveness, cost, and real-world implementation.

## Introduction

Kidney stone disease (KSD) is an increasing global health concern, with its incidence and prevalence rising over the past four decades. As a recurring condition, KSD can necessitate long-term management strategies to prevent recurrence and enhance patient outcomes [1, 2]. Dietary and lifestyle modifications are central to prevention, with key strategies including increased fluid intake and dietary interventions tailored to stone composition [3–5].

Hydration is particularly critical, with guidelines recommending a urine output of at least 2–3 L per day to prevent supersaturation of stone-forming solutes [3, 6, 7]. Patients are also advised to limit dietary sodium, moderate their intake of animal protein, and incorporate plant-based foods rich in protective nutrients, such as citrate, potassium, and magnesium [6–8]. Additionally, obesity and hyperinsulinemia contribute to stone formation through lower urinary pH and increased urinary excretion of calcium, oxalate, and uric acid [9]. Given these multifactorial risks, maintaining a healthy BMI through lifestyle and dietary interventions is essential for reducing recurrence [10]. However, long-term adherence to these recommendations remains a significant challenge for many patients.

Mobile health (mHealth) applications (apps) have emerged as valuable tools in chronic disease management, offering accessible, personalised, and cost-effective healthcare solutions [10]. These digital platforms support patients through education, self-monitoring, and tailored recommendations, improving adherence to treatment plans. Studies have shown that mHealth apps enhance self-efficacy and patient engagement, leading to improved outcomes in conditions such as diabetes, hypertension, and kidney disease [11]. By facilitating self-management and improving clinician-patient communication, mHealth interventions have the potential to bridge existing gaps in traditional healthcare delivery [12].

Despite the growing role of mHealth in chronic disease care, little is known about the specific needs and preferences of patients with kidney stones regarding smartphone-based support tools. To address this gap, we conducted a survey to assess which app features patients with kidney stones would find most beneficial. This study aims to provide insights into patient priorities for digital interventions, guiding the development of tailored mHealth solutions to support kidney stone prevention and management.

## Methods

### Study design and participants

This study employed a quantitative, cross-sectional design using data from a bespoke survey. The survey was conducted between January 2024 and May 2024 and was distributed to patients with recurrent kidney stone disease (KSD) attending the outpatient urology clinic at Mercy University Hospital. Ethical approval was obtained from the Cork Research Ethics Committee (CREC). Recruitment was conducted using convenience sampling over five months. Data is reported in line with STROBE guidelines. A formal power calculation was not undertaken as this was designed as an exploratory pilot survey.

### Inclusion criteria

Participants were required to be adults aged 18 years or older with a confirmed diagnosis of recurrent KSD (one or more recurrences), defined as at least one previous stone episode requiring intervention. They also had to be receiving outpatient follow-up care for KSD. These criteria ensured that participants had direct experience managing recurrent KSD and were actively engaged in outpatient treatment, making them suitable respondents for the mobile health application preferences survey. Eligible patients were provided with printed or digital access to the survey and could complete it in the waiting area or at any point during their clinic visit. Participation was entirely voluntary, and patients could decline without any impact on their clinical care. To maintain confidentiality, surveys were completed anonymously.

### Exclusion criteria

Individuals who did not have a confirmed diagnosis of KSD or had only experienced a single episode of KSD without intervention and no recurrence were excluded from the study. Patients who were not receiving outpatient follow-up care for KSD were also excluded. Additionally, individuals under the age of 18 were not eligible to participate.

### Data collection

Data were collected through an anonymous, self-administered questionnaire distributed to eligible participants during clinic visits. The survey gathered demographic information, including age, gender, and other relevant characteristics. Patient concerns about future kidney stone formation were assessed using a Likert scale ranging from 1 (not concerned) to 5 (very concerned). Interest in a kidney stone management mobile application was also evaluated using a Likert scale from 1 (very unlikely) to 5 (very likely). Additionally, participants were asked about their preferences for potential app functionalities, including tracking fluid intake, receiving dietary recommendations, monitoring dietary intake, and accessing educational resources.

### Statistical analysis

Descriptive statistics were used to summarise demographic characteristics, patient concerns, and interest in the mobile application. Categorical variables were analysed using frequencies and percentages, while continuous variables were reported as means with standard deviations. Chi-Square test was used to determine associations between categorical variables. Cramér’s V test was used to assess the strength of association between two categorical variables. Multivariable analysis was not performed due to the small sample size. Statistical analyses were performed using Qualtrics (Qualtrics International Inc., Provo, UT, USA).

## Results

### Patient characteristics

This cohort consisted of 55 patients, comprising 32 males (58.2%), 22 females (40.0%), and one non-binary individual (1.8%). The age distribution was as follows: 18–24 years (*n* = 7, 12.7%), 25–34 years (*n* = 1, 1.8%), 35–44 years (*n* = 14, 25.5%), 45–54 years (*n* = 17, 30.9%), 55–64 years (*n* = 8, 14.5%), and ≥ 65 years (*n* = 8, 14.5%). The frequency of kidney stone episodes varied among patients: 19 individuals (34.5%) reported a single occurrence, 11 (20.0%) had two episodes, 13 (23.6%) experienced three episodes, 4 (7.3%) had four episodes, 3 (5.5%) had five episodes, and 5 (9.1%) reported six or more episodes.

### Use of mHealth applications

Participants reported varying usage patterns of mobile health (mHealth) applications. Sixteen (29.1%) used health-related apps daily, 7 (12.7%) used them weekly, 7 (12.7%) used them monthly, and 5 (9.1%) used them yearly. Notably, 20 participants (36.4%) had never used a health-related mobile application.

### KSD prevention and risk awareness

**Fig. 1:**
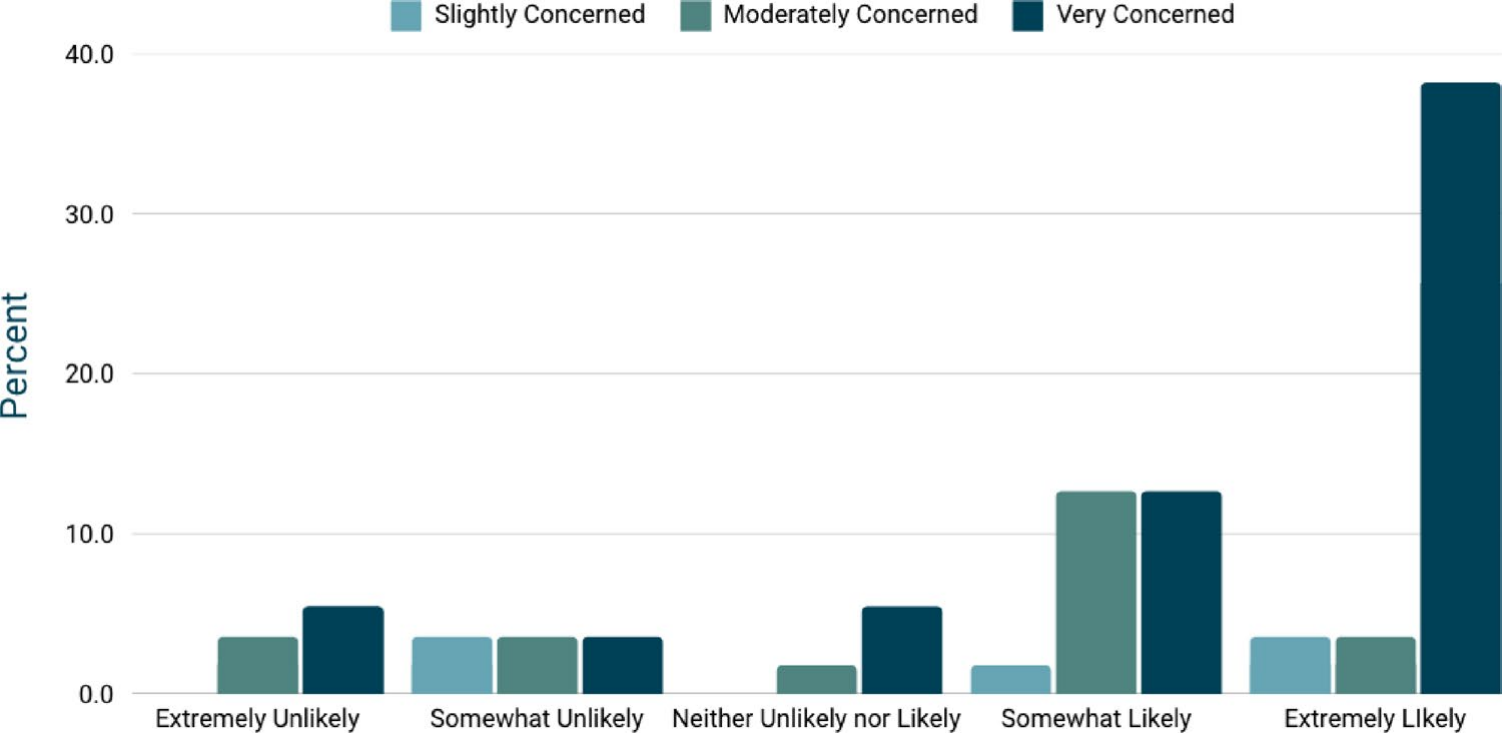
Relationship between patient concern about preventing kidney stones and their likelihood of using a self-monitoring app. A chi-square test (*p* = 0.00425) indicates a strong statistically significant association, with a large effect size (Cramér’s V = 0.582). Patients who are more concerned about kidney stone prevention are significantly more likely to use the app, highlighting the demand for a digital self-monitoring tool tailored to dietary management and prevention strategies

Seventeen participants (30.9%) reported being familiar with risk factors for kidney stone formation, while 34 (61.8%) had some knowledge but sought further information. Four participants (7.3%) were unaware of contributing factors. When assessing levels of concern about kidney stone prevention, 5 participants (9.1%) expressed slight concern, 14 (25.5%) expressed moderate concern, and 36 (65.5%) expressed very high concern. Statistical analysis demonstrated a significant association between participants’ level of concern about kidney stone prevention and their likelihood of using the app, *p* < 0.01 (Fig. 1).

### Likelihood of using a KSD prevention app

**Fig. 2:**
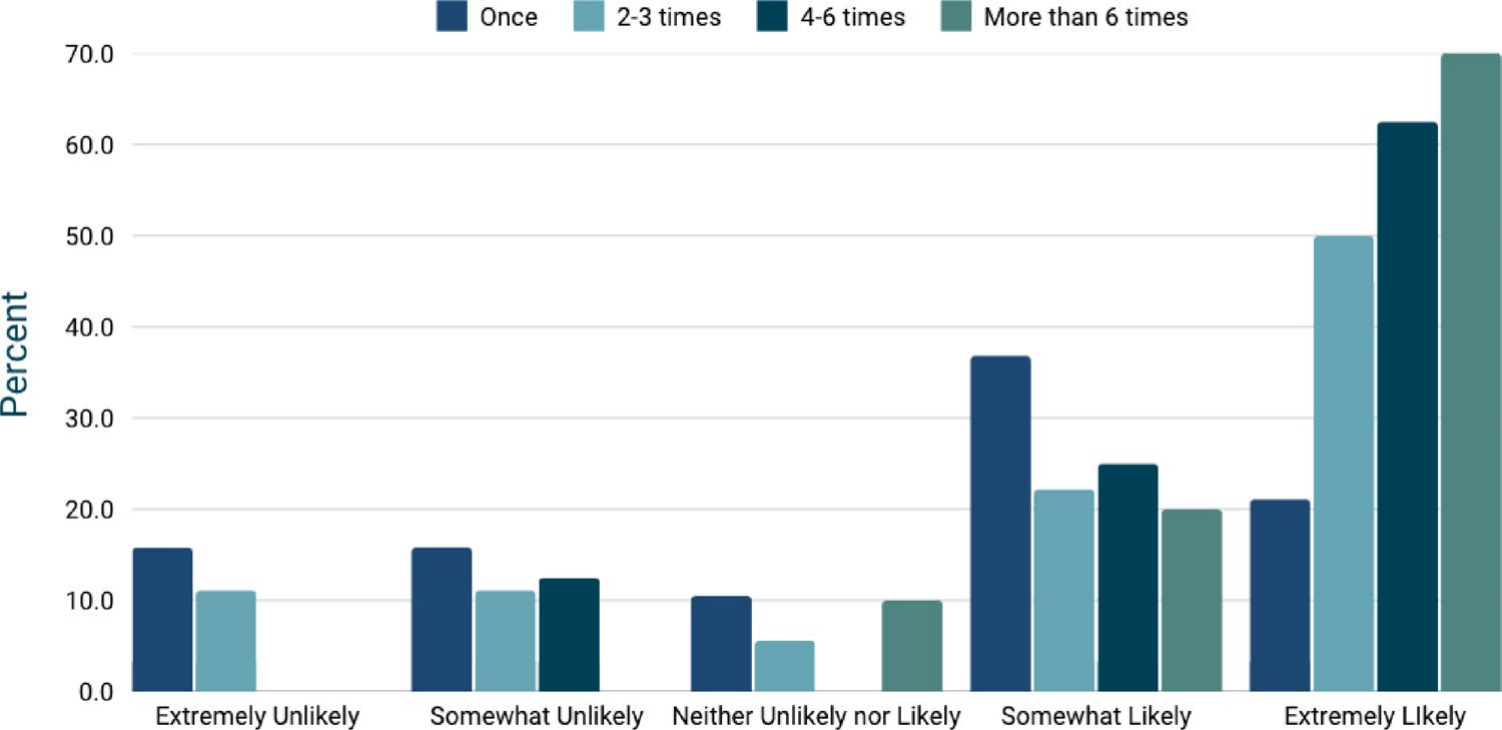
Relationship between the number of kidney stone diagnoses and a patient’s likelihood of using a self-monitoring app. A chi-square test, *p* = 0.503) indicates no statistically significant association, with a small effect size (Cramér’s V = 0.259). This suggests that the frequency of previous kidney stone episodes does not strongly influence a patient’s decision to use the app, highlighting the need to consider other motivating factors, such as perceived health benefits and preventive awareness, when designing and promoting the tool

Participants expressed varied interest in using a kidney stone prevention app. Five (9.1%) were extremely unlikely to use the app, 6 (10.9%) were somewhat unlikely, 4 (7.3%) were neutral, 15 (27.3%) were somewhat likely, and 25 (45.5%) were highly likely to use the app. However, no significant relationship was found between the number of prior kidney stone diagnoses and the likelihood of using the app (*p* = 0.50), as shown in Fig. 2.

### Preferred features of the KSD prevention app

Participants identified several key features they would find beneficial in a kidney stone prevention app. The most frequently selected features included a daily water intake tracker (81.1%), dietary modification recommendations (77.4%), educational content about kidney stones (56.6%), a diet tracker (56.6%), progress tracking and reports (45.3%), integration with wearable devices (22.6%), and medication reminders (17.0%). When asked to prioritise a single most important feature, qualitative content analysis revealed that participants emphasised diet recommendations, water tracking, and educational resources as their top priorities. Preferences for educational content delivery varied, with 11 participants (20.0%) preferring articles and blog posts, 11 (20.0%) favouring videos and animations, 7 (12.7%) selecting infographics and visual aids, 1 (1.8%) preferring quizzes and interactive games, and 20 (36.4%) favouring personalised recommendations based on user data.

### Cost preferences and recommending the app

**Fig. 3:**
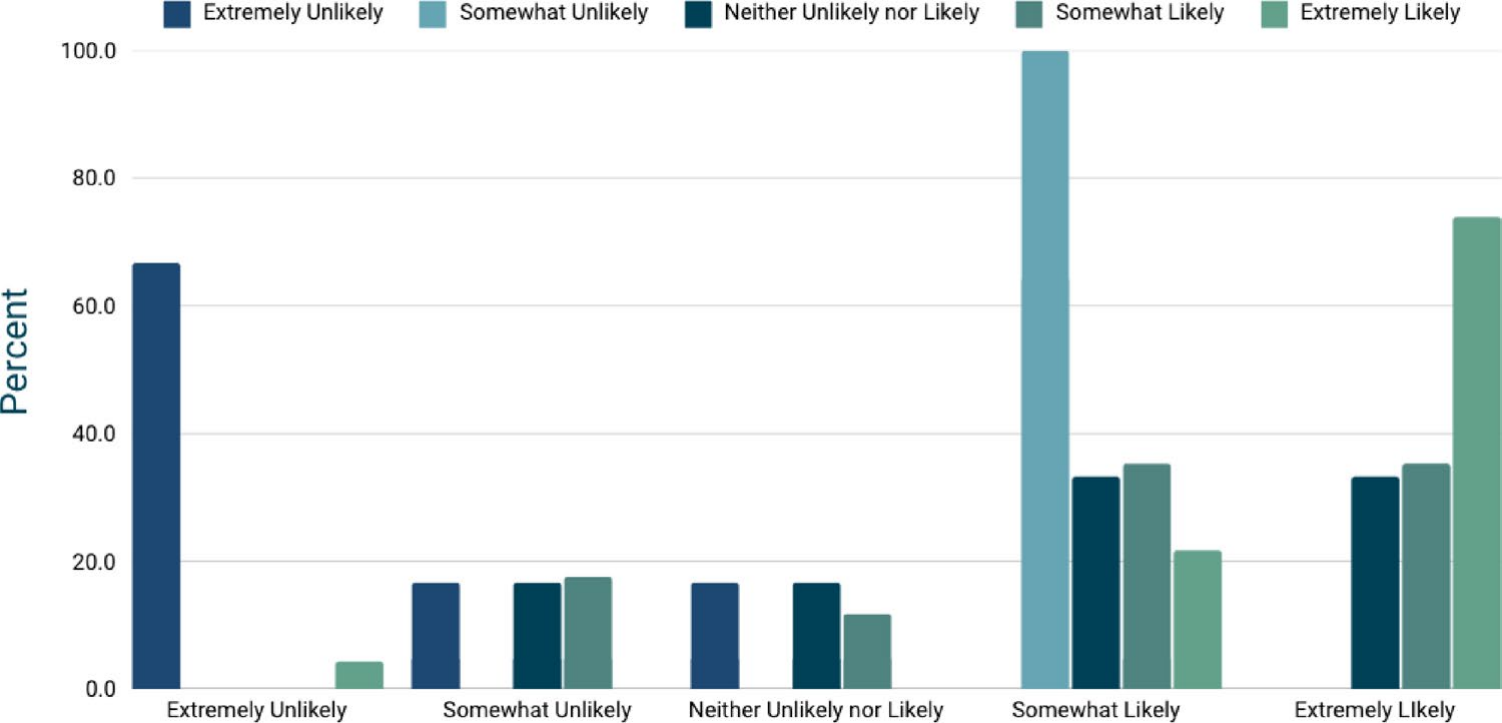
Relationship between a patient’s likelihood of recommending the kidney stone self-monitoring app and their likelihood of using it. A chi-square test, *p* = 0.000120) indicates a strong statistically significant association, with a moderate-to-large effect size (Cramér’s V = 0.458). Patients who are more likely to use the app are also more likely to recommend it, suggesting that perceived utility and personal adoption play key roles in willingness to advocate for the app’s use among peers

Opinions regarding a paid version of the app were divided. Sixteen participants (29.1%) were willing to pay for a premium version, 19 (34.5%) preferred a free version with advertisements, and 18 (32.7%) preferred a free version without ads. Participants also provided insights into their willingness to recommend the app. Six (10.9%) were extremely unlikely to recommend it, 1 (1.8%) was somewhat unlikely, 6 (10.9%) were neutral, 17 (30.9%) were somewhat likely, and 23 (41.8%) were highly likely. A statistically significant association was found between participants’ likelihood of using the app and their likelihood of recommending it to others (*p* < 0.001, effect size = 0.46) (Fig. 3).

## Discussion

The results of this study highlight a high level of interest and perceived utility for a KSD prevention mHealth app. The patients expressed preferences focused on tracking water intake, dietary guidance, and educational content, with notable interest in personalised content delivery and user-friendly interfaces. As expected, those with the most significant concerns about prevention and those who were likely to recommend the app were more inclined to use it. These results underscore the significant relationship between concern about kidney stone prevention and the likelihood of using the app. Targeting individuals with heightened health awareness may enhance the adoption of health-related practices [13]. The correlation between recommending and using the app suggests that user satisfaction and perceived value are key drivers of broader adoption. No significant relationship was observed between the number of prior kidney stone recurrences and app usage, consistent with research suggesting that past experiences do not necessarily increase interest in mHealth solutions [14]. This indicates a need for targeted education to address misconceptions and barriers to adoption.

The results also highlight the potential of mHealth apps for preventing kidney stones. Many patients expressed interest in mobile learning, aligning with broader trends that show an increasing use of health apps [15]. Incorporating water tracking, dietary guidance, and education could significantly enhance self-care, improving patient satisfaction and outcomes [6, 7, 16, 17]. Additionally, targeting those most concerned about kidney stone prevention may boost adoption rates. mHealth apps can also reduce healthcare costs [18]. Widespread adoption of a kidney stone prevention app could minimise emergency visits and surgical interventions. However, engagement barriers, such as high data entry burdens, low motivation, cost and digital health literacy must be addressed [13]. Solutions include digital health literacy education, co-funding or subsidised mHealth devices, tailored reminders, user-friendly interfaces, and reward systems. Integration with wearable devices may further improve convenience.

The findings align with broader trends in the adoption of mHealth. Patients with chronic conditions prefer mobile solutions for disease management due to accessibility and convenience [19]. However, declining user engagement over time and limited clinician involvement remain challenges [13]. Enhancing provider interaction could improve engagement and support behavioural change [20]. Educational modules addressing misconceptions about kidney stones could further improve app utilisation. This study’s emphasis on educational content highlights the need for better dietary counselling. The correlation between recommending and using the app underscores the importance of a positive user experience for broader adoption [14].

This study has several strengths. The survey combined Likert-scale items with free-text responses, capturing both measurable trends and richer insights. The questions were designed around real-world scenarios, enhancing the practical relevance of the findings. Limitations include the modest sample size and single-centre recruitment, which may limit representativeness. Voluntary participation could have favoured more health-conscious or digitally engaged patients, and cultural or socioeconomic factors were not captured [21]. Analyses were limited to descriptive statistics and chi-square tests, which are appropriate for this pilot survey but insufficient for more detailed modelling. Despite these limitations, the study provides valuable insights into patient needs and preferences in kidney stone disease, offering a foundation for developing tailored mHealth interventions. Future research should build on this work through larger multi-centre studies, feasibility testing, and cost-effectiveness analyses.

Future research should address the study’s limitations by including a larger and more diverse population. Exploring personalised features, such as culturally tailored content and adaptive reminders, could enhance appeal and effectiveness. Longitudinal studies evaluating the app’s impact on recurrence rates, healthcare costs, and patient satisfaction would provide further evidence of its value. Given the broader implications of mHealth, this app could serve as a model for similar tools targeting other chronic conditions.

## Conclusion

This pilot survey highlights the potential of a kidney stone prevention mHealth app to enhance patient education, self-management, and health outcomes. By targeting those most concerned about prevention and prioritising user-friendly features, such an app could fill a critical gap in kidney stone care. The findings represent a needs-based assessment to guide app design rather than an evaluation of an existing product. Future research should include larger, multi-centre surveys to confirm these findings, followed by feasibility studies of prototype development, and ultimately randomised controlled trials, longitudinal studies, and cost-effectiveness analyses to establish clinical utility and sustainability.

## Data Availability

Data available on request.
